# Structure of the human histone chaperone FACT Spt16 N-terminal domain

**DOI:** 10.1107/S2053230X15024565

**Published:** 2016-01-22

**Authors:** G. Marcianò, D. T. Huang

**Affiliations:** a Cancer Research UK Beatson Institute, Garscube Estate, Switchback Road, Glasgow G61 1BD, Scotland

**Keywords:** FACT, Spt16, histone chaperone, histones, pita-bread fold, aminopeptidase

## Abstract

The histone chaperone FACT plays an important role in facilitating nucleosome assembly and disassembly during transcription. FACT is a heterodimeric complex consisting of Spt16 and SSRP1. The N-terminal domain of Spt16 resembles an inactive aminopeptidase. How this domain contributes to the histone chaperone activity of FACT remains elusive. Here, the crystal structure of the N-terminal domain (NTD) of human Spt16 is reported at a resolution of 1.84 Å. The structure adopts an aminopeptidase-like fold similar to those of the *Saccharomyces cerevisiae* and *Schizosaccharomyces pombe* Spt16 NTDs. Isothermal titration calorimetry analyses show that human Spt16 NTD binds histones H3/H4 with low-micromolar affinity, suggesting that Spt16 NTD may contribute to histone binding in the FACT complex. Surface-residue conservation and electrostatic analysis reveal a conserved acidic patch that may be involved in histone binding.

## Abstract 2

The Spt16–SSRP1 heterodimer is a histone chaperone that plays an important role in regulating chromatin assembly. Here, a crystal structure of the N-terminal domain of human Spt16 is presented and it is shown that this domain may contribute to histone binding.

## 1. Introduction   

Eukaryotes package their DNA into nucleosomes, which consist of four histone proteins, H2A, H2B, H3 and H4, that associate to form two H2A/H2B heterodimers and one H3/H4 heterotetramer. Two H2A/H2B heterodimers and one H3/H4 heterotetramer associate to form an octamer around which 147 bp of DNA are wrapped (Luger *et al.*, 1997 ▸). Histone chaperones play important roles in directing histone deposition into DNA and histone disassembly from the nucleosome to facilitate DNA replication, transcription and DNA-damage response (Gurard-Levin *et al.*, 2014 ▸).

Facilitating chromatin transcription (FACT) is a histone chaperone consisting of a heterocomplex of suppressor of Ty 16 (Spt16) and structure-specific recognition protein 1 (SSRP1). Early *in vitro* studies showed that FACT displaces the H2A/H2B dimer from the nucleosome template to facilitate RNA polymerase II-driven transcription and has the ability to deposit histones onto DNA (Orphanides *et al.*, 1998 ▸, 1999 ▸). Subsequent studies showed that when FACT is incubated with H2AX and the nucleosome, it can catalyse the exchange of H2AX with H2A within the nucleosome (Heo *et al.*, 2008 ▸) and compete against DNA for H2A/H2B binding but not the H3/H4 tetramer (Winkler *et al.*, 2011 ▸). These studies support a model in which FACT functions as an H2A/H2B exchanger. A recent study has shown that yeast FACT can relax DNA–histone interactions without removing H2A/H2B and the loss of dimer is likely to be owing to reorganization of the destabilized nucleosome (Xin *et al.*, 2009 ▸). Nonetheless, to destabilize the nucleosome, FACT is likely to disrupt DNA–histone interactions by binding directly to the histones. Indeed, early studies showed that FACT and both subunits of FACT can bind histones (Belotserkovskaya *et al.*, 2003 ▸; Formosa *et al.*, 2001 ▸). Moreover, affinity analyses showed that FACT interacts with the core domains and N-terminal tails of both histones H2A/H2B and H3/H4 through several synergistic binding events involving both Spt16 and SSRP1 (Winkler *et al.*, 2011 ▸). How Spt16 and SSRP1 interact with histones remains elusive.

SSRP1 contains an N-terminal Spt16-binding domain followed by a middle domain (MD) and a C-terminal HMG domain. SSRP1 MD adopts two pleckstrin-homology (PH) folds and has been shown to bind histone H3/H4 (VanDemark *et al.*, 2006 ▸; Zunder *et al.*, 2012 ▸). Genetic analysis of yeast SSRP1 MD revealed that some mutations, including Q308K, can induce temperature sensitivity, hydroxyurea sensitivity and an Spt− phenotype, suggesting the importance of this domain in transcription and DNA replication, possibly through interactions with histones (VanDemark *et al.*, 2006 ▸). The HMG domain has been shown to bind nucleosomal DNA and this interaction causes DNA bending, leading to minor-groove widening that may disrupt DNA–histone interactions (Masse *et al.*, 2002 ▸; Kasai *et al.*, 2005 ▸).

Spt16 consists of an NTD followed by an SSRP1 dimerization domain, MD and C-terminal domain. Spt16 MDs from *Chaetomium thermophilum* and *Saccharomyces cerevisiae* have been shown to interact with histone H3/H4, and *C. thermophilum* Spt16 MD also binds H2/H2B (Kemble *et al.*, 2013 ▸; Hondele *et al.*, 2013 ▸). The structure of Spt16 MD reveals two PH domains in tandem and it shares structural similarity with SSRP1 MD and RTT106; however, it has three additional α-helices located at the C-terminus that form a U-turn motif. This motif interacts with the H2A/H2B dimer *via* hydrophobic interactions with residues on the H2B α1 helix (Hondele *et al.*, 2013 ▸). Interestingly, most of the Spt16 mutations that affect FACT activity are in Spt16 MD (Myers *et al.*, 2011 ▸; Formosa *et al.*, 2001 ▸). These mutations can be suppressed by destabilizing H2A/H2B dimer and H3/H4 tetramer interactions (Myers *et al.*, 2011 ▸; McCullough *et al.*, 2011 ▸; Hainer *et al.*, 2012 ▸), suggesting that Spt16 MD is responsible for establishing important interactions with the nucleosome. The Spt16 C-terminal domain consists of highly intrinsically disordered acidic residues. Removal of these regions hampers the ability of FACT to bind nucleosomes and histones and to promote transcription (Belotserkovskaya *et al.*, 2003 ▸; Myers *et al.*, 2011 ▸). A recent structural study showed that the Spt16 and SSRP1 C-terminal flexible acidic residues contain a tyrosine or phenylalanine residue, respectively, that binds to a hydrophobic pocket in H2B (Kemble *et al.*, 2015 ▸). This interaction overlaps with nucleosomal DNA–H2A/H2B interactions, suggesting that FACT might promote reorganization of the nucleosomes by competing with DNA for histone binding.

The function of Spt16 NTD remains unclear. Studies in yeast showed that deletion of this domain or mutations in specific conserved residues do not disrupt essential functions of yeast FACT. However, when combined with the yeast SSRP1 MD Q308K mutation that causes defects in DNA replication and transcription, the effect is lethal, suggesting that Spt16 NTD functionally overlaps with SSRP1 MD (O’Donnell *et al.*, 2004 ▸; VanDemark *et al.*, 2008 ▸). Furthermore, genetic analyses showed that Spt16 NTD might interact with the C-terminal extension of H2A (VanDemark *et al.*, 2008 ▸). The structures of Spt16 NTD from *S. cerevisiae* and *Schizosaccharomyces pombe* (PDB entries 3bip and 3cb5, respectively) reveal an aminopeptidase-like domain that resembles a ‘pita-bread’ fold and which has lost its aminopeptidase activity (Stuwe *et al.*, 2008 ▸; VanDemark *et al.*, 2008 ▸). Studies on *S. pombe* Spt16 NTD showed that it forms a tight complex with histone H3/H4. Moreover, it binds H3 and H4 N-terminal tails with low-micromolar affinity (Stuwe *et al.*, 2008 ▸). In contrast, no binding of histone N-terminal tails could be observed with *S. cerevisiae* Spt16 NTD (VanDemark *et al.*, 2008 ▸). To further elucidate the role of Spt16 NTD in histone interactions, we determined the crystal structure of human Spt16 NTD and examined its histone-binding ability.

## 2. Materials and methods   

### 2.1. Macromolecule production   

The gene for human Spt16 NTD (residues 1–510) was cloned into pGEX-4T-1 vector (Novagen) using standard PCR ligation techniques such that the protein is expressed with an N-terminal glutathione *S*-transferase (GST) tag followed by a TEV protease cleavage site. The resulting vector was transformed into *Escherichia coli* BL21 (DE3) Gold competent cells. The cells were grown at 310 K in LB to an OD_600_ of ∼0.7–0.8 and then induced with 0.2 m*M* isopropyl β-d-1-thiogalactopyranoside overnight at 293 K. The following day, the cells were harvested and resuspended in 50 m*M* Tris–HCl pH 7.6, 0.2 *M* NaCl, 1 m*M* DTT, 2.5 m*M* phenylmethylsulfonyl fluoride, 1 mg ml^−1^ lysozyme and then subjected to a freeze–thaw cycle. The cells were lysed by sonication and then centrifuged at 48 000*g* for 25 min at 277 K using a JA-25.50 rotor (Beckman Coulter) to remove cell debris. GST-Spt16 NTD was purified from the lysate by glutathione Sepharose affinity chromatography. The GST tag was released by incubation with TEV protease and subsequently removed with a glutathione Sepharose column. Cleaved Spt16 NTD was then purified by anion-exchange chromatography (Source Q, GE Life Sciences) in buffer consisting of 50 m*M* Tris–HCl pH 7.6, 1 m*M* DTT, 0–1 *M* NaCl and by size-exclusion chromatography (Superdex 75, GE Life Sciences) in buffer consisting of 25 m*M* Tris–HCl pH 7.6, 150 m*M* NaCl, 1 m*M* DTT. Fractions containing Spt16 NTD were pooled together and concentrated to about 13.3 mg ml^−1^. The protein was then cooled in liquid nitrogen and stored at 193 K. *Gallus gallus* histone octamer proteins (H2A, H2B, H3 and H4) were purified from chicken blood (Lambert *et al.*, 1999 ▸) and separated into histones H2A/H2B and H3/H4 by cation-exchange chromatography (Source S, GE Life Sciences) using a 0–2 *M* KCl gradient in buffer consisting of 50 m*M* KH_2_PO_4_, 50 m*M* K_2_HPO_4_ pH 6.5, 1 m*M* DTT. Proteins were then frozen in liquid nitrogen and stored at 193 K.

### 2.2. Crystallization   

Spt16 NTD (13.3 mg ml^−1^) was mixed with an equal volume of reservoir solution consisting of 0.1 *M* sodium acetate pH 5.0, 17%(*w*/*v*) PEG 3350, 0.1 *M* ammonium iodide using the hanging-drop vapour-diffusion method at 292 K. Crystals appeared after 1 d and were flash-cooled in 0.1 *M* sodium acetate pH 5.0, 17%(*w*/*v*) PEG 3350, 0.1 *M* ammonium iodide, 20%(*w*/*v*) glycerol (Table 1 ▸).

### 2.3. Data collection and processing   

Diffraction data from human Spt16 NTD crystals were collected on beamline I02 at Diamond Light Source (DLS). Data obtained at 100 K were integrated, merged and scaled using the automated *XDS* (Kabsch, 2010 ▸; Winter, 2010 ▸) method applied at the beamline. Data-collection and processing statistics are shown in Table 2 ▸.

### 2.4. Structure-solution and refinement statistics   

Human Spt16 NTD crystals belonged to space group *F*432 with one molecule in the asymmetric unit. Initial phases were obtained by molecular replacement with *Phaser* (Storoni *et al.*, 2004 ▸) using *S. cerevisiae* Spt16 NTD (PDB entry 3biq; 32% sequence identity to human Spt16 NTD; VanDemark *et al.*, 2008 ▸) as the initial search model. The model was built in *Coot* (Emsley & Cowtan, 2004 ▸) and refined using *PHENIX* (Adams *et al.*, 2010 ▸). The structure of human Spt16 NTD was refined to 1.84 Å resolution. The model contains only chain *A* (residues 2–432). Residues 433–510 were absent from the electron density. Side chains with poor electron density were built as stubbed residues. Details of the refinement statistics are shown in Table 3 ▸.

### 2.5. Isothermal titration calorimetry   

Binding interactions between human Spt16 NTD and histones H2A/H2B, H3/H4 or the N-terminal tails of H3 or H4 were analysed by ITC using an iTC200 (MicroCal, GE Life Sciences). Experiments were conducted at 298 K with buffer consisting of 20 m*M* HEPES–NaOH pH 7.5, 150 m*M* NaCl, 1 m*M* TCEP. Histone H2A/H2B or H3/H4 was loaded into the cell at a concentration of 30–40 µ*M*. Human Spt16 NTD was loaded into the injection syringe at a concentration ten times higher than that of the histones in the cell. For interactions of Spt16 NTD with H3 or H4 N-terminal peptides, human Spt16 NTD was loaded into the cell at a concentration of 50 µ*M* while histone H3 or H4 peptides were loaded into the syringe at a concentration 10–20 times higher than that of Spt16 NTD in the cell. 20 injections (2 µl each) were added every 180 s to the cell. For control experiments, buffer was injected into the cell containing the protein. ITC data were generated by subtracting the raw data from the control experiment and were then analyzed using the *Origin* software (v.7). The histone N-terminal peptides sequences are MARTKQTA­RKSTGGKAPRKQLATKAARKSAPSTGG­VKK for H3 (Abgent) and MSGRGKGGKGLGKGGAKRHRKVL­RDN for H4 (Abgent). Peptides were weighed and dissolved in Milli-Q water at 50 m*M* and then diluted into the appropriate buffer.

## 3. Results and discussion   

### 3.1. Crystal structure of human Spt16 NTD   

**Figure 1:**
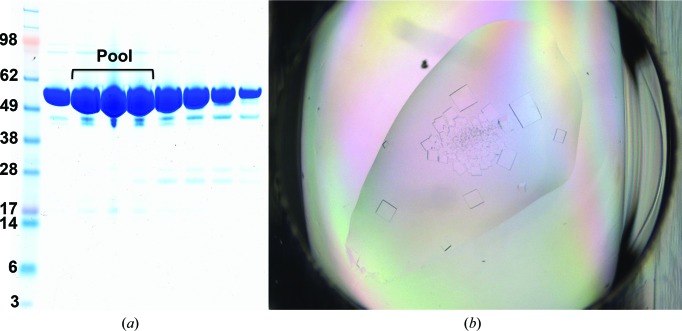
Purity and crystals of human Spt16 NTD. (*a*) SDS–PAGE showing fractions of human Spt16 NTD after Superdex 75 gel-filtration chromatography. (*b*) Crystals of human Spt16 NTD.

**Figure 2:**
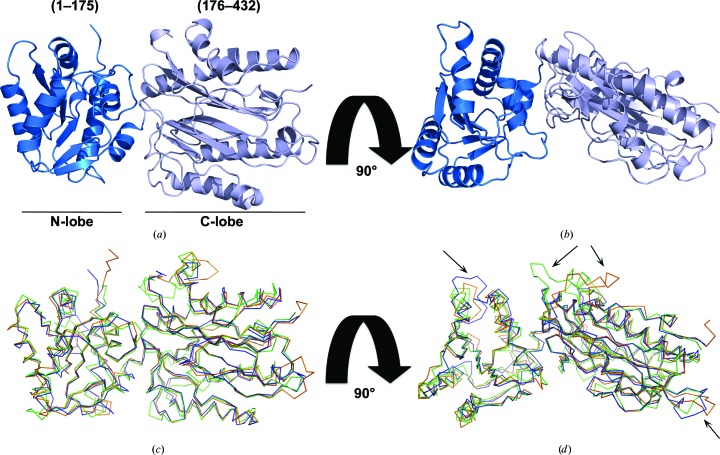
Crystal structure of human Spt16 NTD. (*a*, *b*) Structure of human Spt16 NTD in two orientations. The domain consists of an N-terminal lobe (residues 1–175, blue) and a C-terminal lobe (residues 176–432, light blue). (*c*, *d*) Superimposition of human (blue), *S. cerevisiae* (green; PDB entry 3bip) and *S. pombe* (orange; PDB entry 3cb5) Spt16 NTD structures. (*c*) and (*d*) are displayed in the same orientations as (*a*) and (*b*), respectively. Arrows indicate variations in the loops owing to differences in protein sequence and length.

Our human Spt16 NTD construct consists of residues 1–510. The purified protein gave a band at between 49 and 62 kDa (Fig. 1 ▸ *a*), which agrees with the calculated molecular weight of 57.9 kDa. The protein crystallized at 292 K after 1 d (Fig. 1 ▸ *b*) and these crystals diffracted to a resolution of 1.84 Å with space group *F*432 and one molecule of Spt16 NTD per asymmetric unit. While the construct contains residues 1–510, electron density for residues 433–510 was not visible. The structure of human Spt16 NTD reveals two lobes: an N-lobe comprising residues 1–175 and a C-lobe comprising residues 176–432. Although human Spt16 NTD shares only 32 and 35% sequence identity with the structurally characterized *S. cerevisiae* and *S. pombe* Spt16 NTDs, respectively, it adopts a similar aminopeptidase-like fold in which the C-lobe resembles a pita-bread fold (Figs. 2 ▸ *a* and 2 ▸ *b*; Stuwe *et al.*, 2008 ▸; VanDemark *et al.*, 2008 ▸). Superimposition of the human Spt16 NTD structure onto the structures of Spt16 NTD from *S. cerevisiae* (r.m.s.d. of 1.22 Å for all C^α^ atoms) and *S. pombe* (r.m.s.d. of 0.86 Å for all C^α^ atoms) shows slight differences in the loop regions in the N- and C-lobes owing to variations in the amino-acid sequences and lengths (Figs. 2 ▸ *c* and 2 ▸ *d*). Despite the presence of a linker between the two lobes, the orientation of the two lobes appears to be rigid across the three Spt16 NTD structures.

### 3.2. Human Spt16 NTD binds histone H3/H4 with micromolar affinity   

**Figure 3:**
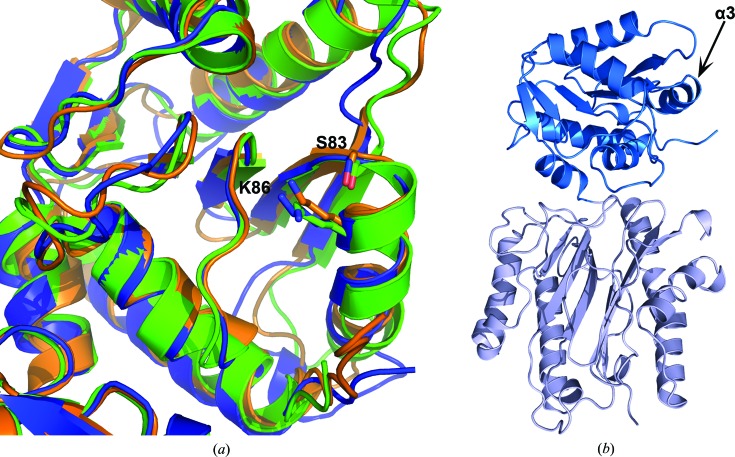
Putative binding site of Spt16 NTD for the H4 N-terminal tail. (*a*) Superimposition of Spt16 NTD structures from human (blue), *S. cerevisiae* (green; PDB entry 3bip) and *S. pombe* (orange; PDB entry 3cb5) showing a close-up view of the H4 N-terminal tail-binding site on the N-lobe. The Ser83 and Lys86 side chains located on the α3 helix on the *S. pombe* Spt16 NTD structure and the corresponding Ser and Lys in human and *S. cerevisiae* Spt16 NTD are indicated. (*b*) Overall structure of human Spt16 NTD showing the location of the α3 helix.

Earlier studies on *S. pombe* Spt16 NTD show that it forms a 1:1 complex with the H3/H4 dimer but that it does not bind the H2A/H2B dimer in pull-down experiments. Moreover, it binds histone H3 and H4 N-terminal tails with low-micromolar affinity (Stuwe *et al.*, 2008 ▸). A pocket harbouring Ser83 and Lys86 in *S. pombe* Spt16 NTD was shown to be involved in binding the H4 N-terminal tail. These residues are conserved in both human and *S. cerevisiae* Spt16 NTD and they adopt a similar configuration in all three structures (Fig. 3 ▸). This prompted us to investigate whether human Spt16 NTD has a role in binding histones and histone N-terminal tails.

**Figure 4:**
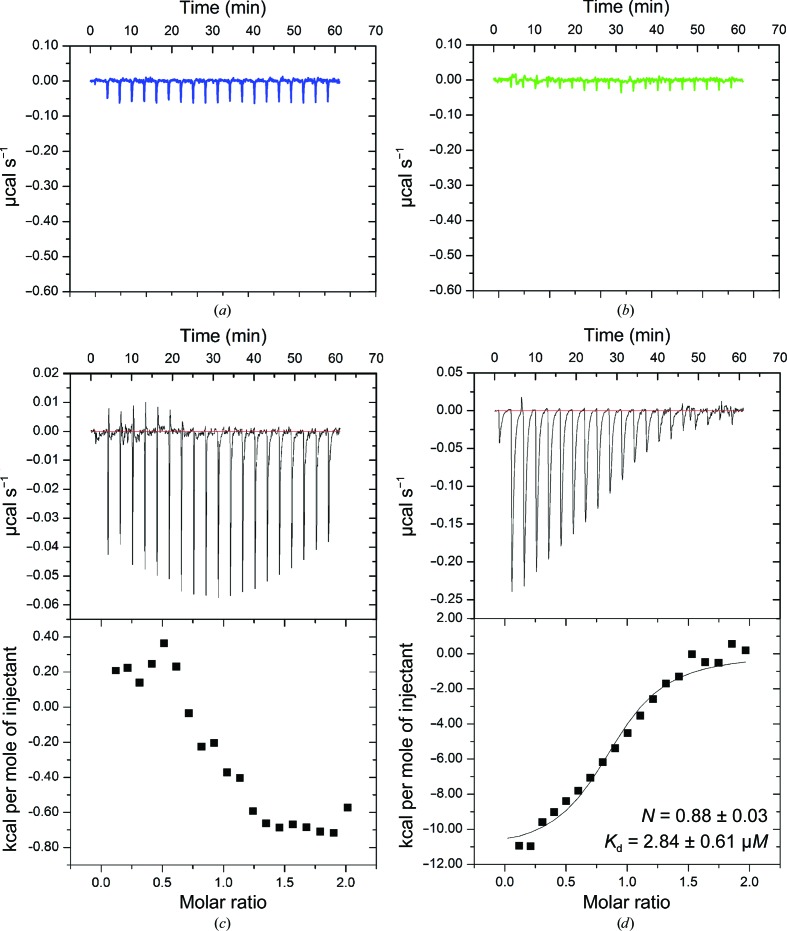
ITC profiles of human Spt16 NTD against (*a*) histone H4 N-terminal tail, (*b*) histone H3 N-terminal tail, (*c*) H2A/H2B and (*d*) H3/H4. (*a*, *b*) Raw data for the N-­terminal peptide from H4 (blue) and H3 (green), respectively, titrated into human Spt16 NTD. (*c*) ITC profile showing raw data (upper panel) and integrated raw data (lower panel) for the titration of human Spt16 NTD against H2A/H2B. (*d*) ITC profile showing raw data (upper panel) and integrated data (lower panel) for the titration of human Spt16 NTD against H3/H4.

**Figure 5:**
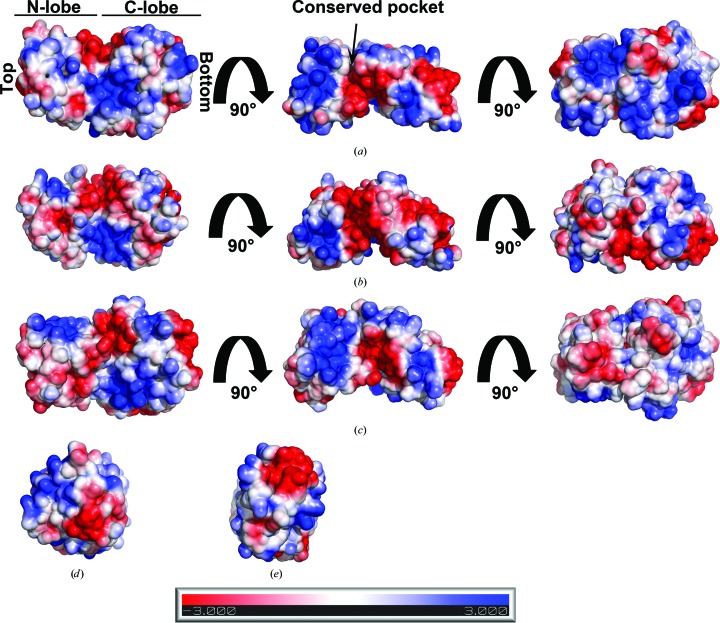
Electrostatic surface potential (−3 to 3*kT*) comparison of human, *S. pombe* and *S. cerevisiae* Spt16 NTD. Electrostatic surface representations of (*a*) human, (*b*) *S. pombe* and (*c*) *S. cerevisiae* Spt16 NTD in different views generated using *APBS* (Baker *et al.*, 2001 ▸) in *PyMOL* (Schrödinger). *S. pombe* Spt16 NTD reveals a larger acidic surface at the conserved pocket than that observed in *S. cerevisiae* and human Spt16 NTD. The left panel is in the same orientation as in Fig. 2 ▸(*a*); the middle and right panels are rotated 90 and 180° about the horizontal axis, respectively. (*d*, *e*) Top and bottom view, respectively, of human Spt16 NTD as shown in the left panel in (*a*). The arrow indicates the location of the conserved pocket.

To assess whether human Spt16 NTD binds the histone H3 and H4 N-terminal tails, we performed ITC experiments to measure the binding affinity between human Spt16 NTD and the H3 or H4 N-terminal peptides. ITC experiments showed that there was no enthalpy change when H3 or H4 peptide was added to human Spt16 NTD (Figs. 4 ▸ *a* and 4 ▸ *b*), suggesting that human Spt16 NTD does not bind histone H3 or H4 N-terminal tails or that the interaction is too weak to be detected at the concentrations used in our assays. It is noteworthy that our assay was performed at 150 m*M* NaCl concentration, while in the studies of *S. pombe* Spt16 NTD 25 m*M* NaCl was used. Stuwe and coworkers have shown that the salt concentration influences the interaction of Spt16 NTD with histone tails *in vitro* (Stuwe *et al.*, 2008 ▸). Unfortunately, human Spt16 NTD precipitated heavily and rapidly at 25 m*M* NaCl, thus preventing further analysis. It is likely that these interactions depend on the ionic strength used in the *in vitro* binding assays and this may differ between species. Another possible explanation that may account for the differences in histone-tail binding abilities across different species is that the human and *S. pombe* Spt16 NTD structures have different electrostatic surface potentials (Fig. 5 ▸). Consistent with this suggestion, no binding of histone N-terminal tails is observed with *S. cerevisiae* Spt16 NTD (VanDemark *et al.*, 2008 ▸).

To examine whether human Spt16 NTD binds histones, we performed ITC analyses of human Spt16 NTD against H2A/H2B or H3/H4. An ITC experiment with human Spt16 NTD against H2A/H2B showed very little change in enthalpy when Spt16 NTD was added to H2A/H2B, suggesting that Spt16 NTD does not bind H2A/H2B or binds with a very weak affinity. On the other hand, human Spt16 NTD binds histones H3/H4 with low-micromolar affinity (*K*_d_ of 2.84 ± 0.61 µ*M*; *N* = 0.88 ± 0.03; Figs. 4 ▸ *c* and 4 ▸ *d*). Together, these data show that human Spt16 NTD is likely to bind H3/H4 *via* the core histone.

### 3.3. A conserved pocket on Spt16 NTD might contribute to histone binding   

**Figure 6:**
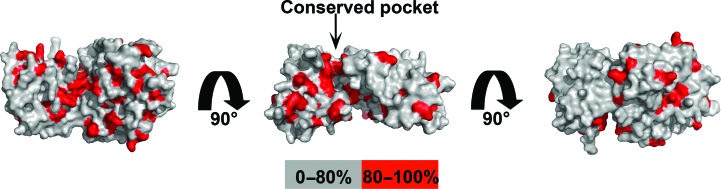
Sequence conservation of Spt16 NTD. Sequence alignment of Spt16 NTD from human, *Danio rerio*, *S. cerevisiae*, *S. pombe*, *Drosophila melanogaster*, *Xenopus laevis*, *Dictyostelium discoideum* and *Chaetomium thermophilum* was performed and the sequence conservation was mapped onto the structure of human Spt16 NTD. (*a*), (*b*) and (*c*) are in the same orientation as in the left, middle and right panels of Fig. 5 ▸(*a*), respectively. The conserved pocket is indicated by an arrow. Residues that are more than 80% conserved are highlighted in red and the rest are coloured grey.

To explore how human Spt16 NTD may interact with histones, we analysed the surface-residue conservation of Spt16 NTD to identify hypothetical patches that may be involved in histone binding. Sequence alignment of Spt16 NTD from various species was performed and the sequence conservation was mapped onto the structure of human Spt16 NTD (Fig. 6 ▸). The analysis revealed clusters of conserved residues on the C-lobe and in a pocket between the N-lobe and the C-lobe. Furthermore, electrostatic surface analysis of human Spt16 NTD showed an acidic patch that co-localizes within this conserved pocket (Fig. 5 ▸ *a*, middle panel), suggesting a possible role of this region in histone interaction. The electrostatic maps also show acidic patches at the bottom of the C-lobe and at the top of the N-lobe that may be potential histone-binding sites (Figs. 5 ▸ *d* and 5 ▸ *e*). Further studies are required to elucidate how Spt16 NTD interacts with histones. Our data are consistent with previous studies suggesting that Spt16 NTD is likely to play a supportive role with other FACT domains in modulating nucleosome assembly.

## 4. Conclusion   

Little is known about Spt16 NTD and its functions. Here, we have reported the crystal structure of human Spt16 NTD and shown that it adopts a structure similar to those of *S. cerevisiae* and *S. pombe* Spt16 NTDs. Our ITC analyses showed that human Spt16 NTD is likely to bind histones H3/H4 through the histone core. Analysis of Spt16 NTD sequences from various species and the electrostatic surface of this domain reveal the presence of a conserved acidic pocket that may be involved in histone binding (Fig. 5 ▸ *a*, middle panel, and Fig. 6 ▸ *b*). Interestingly, some Spt16 NTD mutations that are lethal in conjunction with SSRP1 mutations cluster in a region near this conserved pocket (VanDemark *et al.*, 2008 ▸). Further studies will be required in order to elucidate how FACT domains cooperate to bind histones.

## Supplementary Material

PDB reference: N-terminal domain of human Spt16, 5e5b

## Figures and Tables

**Table 1:** Crystallization

Method	Hanging-drop vapour diffusion
Plate type	24-well hanging-drop plate
Temperature (K)	292
Protein concentration (mg ml^−1^)	13.3
Buffer composition of protein solution	25 m*M* Tris–HCl pH 7.6, 150 m*M* NaCl, 1 m*M* DTT
Composition of reservoir solution	0.1 *M* sodium acetate pH 5.0, 17%(*w*/*v*) PEG 3350, 0.1 *M* ammonium iodide
Volume and ratio of drop	2 µl, 1:1 ratio
Volume of reservoir (µl)	0.5

**Table 2:** Data collection and processing Values in parentheses are for the outer shell.

Diffraction source	DLS beamline I02
Wavelength (Å)	0.979493
Temperature (K)	100
Detector	Pilatus 6M
Crystal-to-detector distance (mm)	264.6
Rotation range per image (°)	0.85
Total rotation range (°)	90
Exposure time per image (s)	0.5
Space group	*F*432
*a*, *b*, *c* (Å)	246.57, 246.57, 246.57
α, β, γ (°)	90, 90, 90
Mosaicity (°)	0.13
Resolution range (Å)	87.18–1.84 (1.88–1.84)
Total No. of reflections	1124024 (72859)
No. of unique reflections	56315 (4094)
Completeness (%)	100.0 (100.0)
Multiplicity	20.0 (17.8)
〈*I*/σ(*I*)〉	28.1 (4.2)
*R*_r.i.m._ †	0.084 (0.822)
*R*_p.i.m._	0.019 (0.193)
Overall *B* factor from Wilson plot (Å^2^)	22.67

† Estimated *R*_r.i.m._ = *R*_merge_[*N*/(*N* − 1)]^1/2^, where *N* is the data multiplicity.

**Table 3:** Structure-solution and refinement statistics Values in parentheses are for the outer shell.

Resolution range (Å)	61.64–1.84 (1.88–1.84)
Completeness (%)	100.0
No. of reflections, working set	53453 (2634)
No. of reflections, test set	2857 (130)
Final *R*_cryst_	0.156 (0.203)
Final *R*_free_	0.176 (0.215)
No. of non-H atoms	
Protein	3420
Solvent	458
Total	3878
R.m.s. deviations	
Bonds (Å)	0.008
Angles (°)	1.216
Average *B* factors (Å^2^)	
Protein	28.4
Ramachandran plot	
Most favoured (%)	98.9
Allowed (%)	100
